# The Complete Mitochondrial Genome and Expression Profile of Mitochondrial Protein-Coding Genes in the Bisexual and Parthenogenetic *Haemaphysalis longicornis*

**DOI:** 10.3389/fphys.2019.00982

**Published:** 2019-07-30

**Authors:** Tianhong Wang, Shiqi Zhang, Tingwei Pei, Zhijun Yu, Jingze Liu

**Affiliations:** Hebei Key Laboratory of Animal Physiology, Biochemistry and Molecular Biology, College of Life Sciences, Hebei Normal University, Shijiazhuang, China

**Keywords:** *Haemaphysalis longicornis*, mitochondrial genome, bisexual and parthenogenetic, mitochondrial protein-coding genes, expression profile

## Abstract

The tick *Haemaphysalis longicornis* is widely distributed in eastern Asia, New Zealand and Australia, and is well-known as a vector of multiple zoonotic pathogens. This species exhibits two reproductive strategies, bisexual and obligate parthenogenetic reproduction. Hence, in the current study, the complete mitochondrial genomes of the bisexual and parthenogenetic populations were assembled and analyzed, and the expression of the mitochondrial protein-coding genes was evaluated and compared between the two reproductive populations. The results indicated that the length of the mitochondrial genomes of the two reproductive populations is 14,694 and 14,693 bp in the bisexual and parthenogenetic populations, respectively. The AT content in the mitochondrial genome of the bisexual and obligate parthenogenetic population reached 77.22 and 77.34%, respectively. The phylogenetic tree was constructed combining 13 protein-coding genes, which showed that the genetic distance between the bisexual and parthenogenetic populations was less than that between the subspecies. The expression of the mitochondrial protein-coding genes was quantitatively analyzed at different feeding status for the bisexual and parthenogenetic populations, and the results showed significant differences in the expression patterns of these genes, suggesting that they might trigger specific energy utilization mechanisms due to their different reproductive strategies and environmental pressures.

## Introduction

Ticks are obligate blood-sucking ectoparasites with global distribution, and they can feed on a broad range of animals (Kaufman, 2010). They are notorious vectors of zoonotic pathogens. Furthermore, tick-borne diseases (TBDs) are increasingly threatening animal and human health and thus causing great economic damages (Jongejan and Uilenberg, 2004; Kovalev and Mukhacheva, 2013). The global annual financial losses due to ticks and TBDs are estimated in billions of dollars (Burger et al., 2014b). In China, 117 species of ticks in 7 genera have been recorded, and more than 60 tick species have shown vector potential (Yu et al., 2015). The tick *Haemaphysalis longicornis* is widely distributed in eastern Asia, New Zealand and Australia and is well-known as a vector of multiple zoonotic pathogens (Hoogstraal et al., 1968; Herrin and Oliver, 1974). These pathogens include *Anaplasma bovis*, *Babesia ovate*, *Borrelia burgdorfer*, *Rickettsia japonica*, *Theileria orientalis*, and severe fever with thrombocytopenia syndrome virus (SFTSV) (Fujisaki et al., 1994; Jongejan and Uilenberg, 2004; Matsuo et al., 2013; Takahashi et al., 2014; Qin et al., 2018; Zhang et al., 2019). Human infection of tick *H. longicornis* was reported in at least 23 provinces in China (Chen et al., 2010, 2015; Zhan et al., 2017) with a case fatality rate of 10−30% (Liu et al., 2014). In America, this species has been expanded to 19 counties in 8 states since the first detection in 2013 (Magori, 2018).

In *H. longicornis*, the life history of a bisexual and parthenogenetic population is different (Hoogstraal et al., 1968). The parthenogenetic population can feed engorged and oviposit without males and thus might have stronger reproductive capacity and spreading ability. However, the distribution of the parthenogenetic population was only reported in Shanghai, Gansu and Sichuan provinces in China (Zhou Z. et al., 2004; Yang et al., 2007; Chen et al., 2010). The parthenogenetic species were historically a critical problem for early taxonomic studies (Monis et al., 2002). The number of eggs laid by the parthenogenetic population was significantly lower than that of the bisexual population (Oliver, 1977), whereas the engorgement body weight of females and egg size of the parthenogenetic population were considerably higher than that of the bisexual population, but the hatching rate of eggs was still lower in the parthenogenetic population (Chen et al., 2014). In recent years, scanning electron microscopy (SEM) has been used to describe the morphological characteristics of the two reproductive populations (Yang et al., 2007; Wang et al., 2013).

Mitochondrial genomes are characterized by simple structure, small molecular weight, rapid evolutionary rate, and matrilineal inheritance, which is particularly crucial in phylogenetic studies (Lin and Danforth, 2004; Gissi et al., 2008; Li and Liang, 2018). To date, more than 20 complete mitochondrial genomes in ixodid ticks have been available for phylogenetic analysis (Burger et al., 2014b). Additionally, structural genomic features, such as secondary structures of tRNA and rRNA, are also applied in comparative and evolutionary genomics (Qin et al., 2015; Kim et al., 2017). Compared with a phylogenetic analysis of a single gene sequence, a combined study of multiple mitochondrial genes can more accurately evaluate the genetic distance among or within species (Papanicolaou et al., 2008; Timmermans et al., 2014). Hence, investigations on the genetic relatedness of mitochondrial genomes will be helpful in making taxonomic determinations. Additionally, the protein-coding genes (PCGs) play a vital role in activity changes of the arthropod mitochondrial complex (Uno et al., 2004; Fontanillas et al., 2010). Evaluation of the differential expression profiling of PCGs will help to elucidate the energy utilization of different reproductive strategies.

In the current study, the complete mitochondrial genomes of the bisexual and parthenogenetic populations of *H. longicornis* were assembled and analyzed, and the expression of the mitochondrial PCGs was evaluated and compared between the two reproductive populations. The differences in survival climate may have influenced the survival strategies of *H. longicornis* and may have resulted from mutations in the parthenogenetic population. These results may help to elucidate the possible interconnections among environmental stress, genetic evolution, and parthenogenetic patterns.

## Materials and Methods

### Sample Collection and DNA Extraction

The bisexual population of *H. longicornis* was collected from Xiaowutai National Nature Reserve Area of Zhuolu County (40^∘^ 03′ 03^″^ N, 115^∘^ 23′ 15^″^ E), Zhangjiakou City, Hebei Province, China. The parthenogenetic population was collected in Cangxi County (31^∘^ 44′ 35^″^ N, 105^∘^ 49′ 04^″^ E), Guangyuan City, Sichuan Province, China. The free-living nymphal collection was conducted in the above two locations using a white cloth flag in April of each year. The collected ticks were placed into perforated, clean centrifugal tubes with ventilated lids on one side through a 4-mm-diameter hole sealed with plastic screen. After the nymphs molted, delimitation between the two populations was performed based on our previous publications (Yang et al., 2007; Chen X. et al., 2012; Chen Z. et al., 2012; Wang et al., 2013). The distinguishing feature between the two populations is whether males appear in the adult population after molting nymphs.

The ticks were fed on the New Zealand white rabbit ear until engorged and were maintained under standard environmental chamber conditions (26 ± 1^∘^C, 75% ± 5 RH, and 12 h: 12 h L:D). All experimental procedures in this study were approved by the Animal Ethics Committee of the Hebei Normal University (Protocol Number: IACUC-157026).

Three mitochondrial genome samples were sequenced as follows: 10 unfed adults of parthenogenetic female, bisexual female and bisexual male were separately placed in a 1.5-ml centrifuge tube, washed with 75% ethanol for 30 s, and then homogenized under liquid nitrogen. DNA was extracted using the EasyPure^®^ genomic DNA kit (TransGen Biotech Co., Ltd., Beijing, China). DNA concentration was estimated using a TU-1950 spectrophotometer (Xi’an Yima Opto-electrical Technology Co., Ltd., Xi’an, China). The extracted DNA was visualized on 1% agarose gel to ensure strong bands and purified using the EasyPure^®^ quick gel extraction kit (TransGen Biotech Co., Ltd., Beijing, China). All purified DNA was stored at −80^∘^C until use.

### Next Generation Sequencing Library Construction and Sequence Analysis

For the sequencing library construction, the fragmented tick genomic DNA (400−500 bp) was obtained by sharing 2-μg of tick DNA using Covaris M220 Focused-ultrasonicator (Convaris, Inc., Woburn, MA, United States). Subsequent reaction steps using TruSeq^TM^ DNA Sample HT Prep Kit (Illumina Inc., San Diego, CA, United States), which include repaired ends, adenylated 3′ ends, added A-Tailing and ligated adapter, and then the ligation products were purified with agarose electrophoresis and enriched the DNA fragments with a PCR primer cocktail that annealed to the ends of the adapters. Finally, the library was quantified and sequenced on illumina Hiseq X Ten sequencing platform according to the standard operation.

In this study, we annotated our next-generation sequencing (NGS) data with the mitochondrial genome of three different species of *Haemaphysalis* from the National Center of Biotechnology Information (NCBI) nucleotide database, as the Genebank number: AB075954 (*H. flava*), JX573135 (*H. formosensis*) and JX573136 (*H. parva*). SOAPdenovo v2.04^1^ was used for sequence filtering and assembly (Xie et al., 2014). GapCloser v1.12 software (a SOAP suite tool) was used to perform vulnerability completion and base correction. The splicing of the original mitochondrial genome sequencing data was reflected in the (Supplementary Table S1). The mitochondrial circular map was assembled by Organellar Genome DRAW^2^ (Lohse et al., 2013). The three complete mitochondrial genome circular maps of *H. longicornis* were uploaded to the (Supplementary Figures S1−S3). The complete mitochondrial genome sequence was deposited in the NCBI nucleotide database accession numbers: MK450606 (The bisexual population of *H. longicornis*) and MK439888 (The parthenogenetic population of *H. longicornis*).

The PCGs, tRNA, rRNA and non-coding regions (NCRs) of the genome were predicted using MITOS^3^ online analysis software (Bernt et al., 2013). The PCGs were blasted using the GO^4^ database. MEGA v7.0 for Windows (Kumar et al., 2016) was used to analyze the base content and the similarity. The calculation formula of the base skew is AT skew = (A−T)/(A+T); GC skew = (G−C)/(G+C) (Alexandre et al., 2005). The relative synonymous Codon usage (RSCU) analysis was carried out using Codon W software version 2.7.2.1 for Windows (Cancilla et al., 1995). The tRNAscan-SE^5^ was used to identify the tRNA structure (Burger et al., 2014a). The genome tandem repeats of NCRs were predicted using Tandem Repeats Finder^6^ (Benson, 1999).

### Polymorphism Detection and Circular Map Comparison

Based on our previous studies (Chen X. et al., 2012; Chen et al., 2015), we speculated that the differentiation of the bisexual population might generate the parthenogenetic population. In this experiment, we analyzed the mitochondrial genome data of the bisexual population as the treatment group to assess the polymorphism and small insertion-deletion of the parthenogenetic population. Burrows-Wheeler Aligner^7^ and Genome Analysis Toolkit^8^ were used to match the genome sequence, and nucleotide polymorphism analysis (Lines et al., 2014; Mccormick et al., 2015). OrthoMCL DB^9^ was used to draw the three circular maps of the mitochondrial genomes (conditions: *E*-value: 1−5, E percent identity cutoff: 0, Markov plus index: 1.5) (Fischer et al., 2011).

### Phylogenetic Development and Homologous Gene

The mitochondrial genomes of 24 ixodid ticks and *Nuttalliella namaqua* were obtained from the NCBI^10^ nucleotide databases. RAxML v8.0^11^ was used to construct a phylogenetic tree with bootstrap replicated evaluation nodes 1000 times (Stamatakis, 2015). Sequence alignments and filtering of the PCGs were carried out using MAFFT v7.0^12^ and Gblocks^13^. A Venn diagram of homologous genes was drawn using CGV software (Tominski et al., 2009).

### Mitochondrial Genome Protein-Coding Genes at Different Feeding Status

The expression profiling of the mitochondrial genome protein-coding genes of *H. longicornis* at different feeding status was quantitatively analyzed using real-time quantitative PCR (qPCR). The weights of the bisexual and parthenogenetic population samples were similar in this experiment. RNA was extracted using the EasyPure^®^ RNA Kit (TransGen Biotech Co., Ltd., Beijing, China) from *H. longicornis* at different feeding status, including unfed female (without feeding), partially fed (feed without mating) and engorged (fully engorged and detached).

Then, cDNA was synthesized using 500-ng RNA and 10-μl purity water combined with TranScript^®^ First-Strand cDNA Synthesis SuperMix (TransGen Biotech Co., Ltd., Beijing, China). Samples were incubated at 65^∘^C for 5 min, chilled on ice for 2 min, and then incubated at 42^∘^C for 15 min. The cDNA products were stored at −20^∘^C. The length of the quantitative product was designed between 100 and 200 bp (Supplementary Table S2), and a 20-μl master mix was prepared using SYBRGreen PCR buffer, HotStarTaq DNA polymerase, SYBRGreen I dye, dNTPs (TransGen Biotech Co., Ltd., Beijing, China), PCR stabilizer. PCR was performed using SimpliAmp Thermal Cycler A24811 (Applied Biosystems Shanghai Co., Ltd., Shanghai, China). The qPCR conditions were set as follows: 95^∘^C for 30 s; 40 cycles of 95^∘^C for 5 s, 55^∘^C for 15 s, 72^∘^C for 10 s. Relative expression of these genes was calculated using the 2^–ΔΔCt^ method. If the relative expression ratio was <1, there was down regulation of the comparison gene in the sample (dotted line group). Conversely, if the expression ratio was >1, there was up regulation in the expression of the sample (dotted line group). All samples were prepared from at least two female individuals, and three parallel experiments were performed to improve the accuracy. The statistical analysis was determined using Duncan’s multiple range test with analysis of variance (ANOVA), and calculations were performed using SPSS v17.0 for Windows (SPSS Inc., Chicago, IL, United States).

## Results

### Base Features and Gene Composition

The length of the complete genome of the bisexual and parthenogenetic populations *H. longicornis* is 14,694 and 14,693 bp, respectively. The circular structure of the mitochondrial genomes of the two populations was successfully predicted, and the gene arrangement of the mitochondrial genomes included 13 PCGs, 22 tRNA and 2 rRNA. The contents of the four bases in the base composition of the mitochondrial genome are approximately 38% (A), 39% (T), 13% (C), and 9% (G), respectively. The A+T contents of the mitochondrial genome account for 77.22% in the bisexual population and 77.34% in the parthenogenetic population. The frequency of base used in both reproductive populations was highly similar. The GC-skew and AT-skew of the two reproductive populations were negative; GC-skew in the bisexual population was −0.1446, and AT-skew in the bisexual population was −0.0104. In the parthenogenetic population, GC-skew was −0.1502, and AT-skew was −0.0092 (Table 1).

**TABLE 1:** Base composition and skewness in the mitochondrial genome of *Haemaphysalis longicornis.*

**Populations**	**A**	**Ratio%**	**T**	**Ratio%**	**C**	**Ratio%**	**G**	**Ratio%**	**AT-skew**	**GC-skew**	**CG%**	**All length**
Female (Bisexual)	5614	38.21%	5732	39.01%	1916	13.04%	1432	9.75%	−0.0104	−0.1446	22.78%	14694
Male (Bisexual)	5614	38.21%	5732	39.01%	1916	13.04%	1432	9.75%	−0.0104	−0.1446	22.78%	14694
Parthenogenetic	5629	38.31%	5734	39.03%	1915	13.03%	1415	9.63%	−0.0092	−0.1502	22.66%	14693

### Polymorphism and Circular Map of the Mitochondrial Genome

**FIGURE 1:**
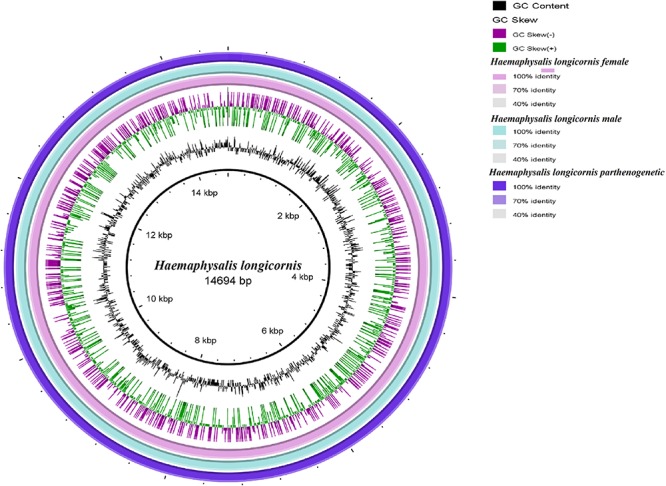
Circular maps of the mitochondrial genome of the bisexual and parthenogenetic *Haemaphysalis longicornis*. The color change of the ring indicates the level of difference between the bisexual female, bisexual male, and the parthenogenetic population.

Bisexual female and male ticks share the same mitochondrial genome. Polymorphism analysis of the parthenogenetic population revealed 199 bases differences, but no small indel differences were found (Supplementary Figure S4). Only one site on the *nad3* gene displayed a non-synonymous mutation. The circular maps of the mitochondrial genomes were assembled and compared. No significant differences in gene direction and arrangement were observed between the bisexual and parthenogenetic populations (Figure 1).

### Mitochondrial Protein-Coding Gene and Codon Analysis

The length of the PCGs in the bisexual population was 10,434 bp, accounting for 71.01% of the total mitochondrial genome, and was 10,464 bp in the parthenogenetic population, accounting for 71.22%. The heavy strand contains 9 genes (*cox1*, *cox2*, *cox3*, *cytb*, *nad2*, *nad3*, *nad6*, *atp6*, and *atp8*), and the light strand contains 4 genes (*nad1*, *nad4*, *nad4l*, and *atp8*). Mitochondrial genes of *H. longicornis* displayed distinct rearrangement characteristics from *Haemaphysalis* genus (Liu et al., 2018), and the arrangement and distribution of 13 PCGs were completely consistent in the two reproductive populations. Four start codons (ATA, ATT, ATG, and ATC) were detected in *H. longicornis*. Among these start codons, ATT was adopted by 7 genes (*cox1*, *cox2*, *nad1*, *nad2*, *nad3*, *nad4l*, and *nad5*), ATA was used by 3 genes (*cox3*, *cytb*, and *atp8*), ATG and ATC were used by 3 genes (*nad4*, *atp6*, and *nad6*). The termination codon TAA was used by 9 PCGs (*cox1*, *cox3, atp6*, *atp8*, *nad1, nad2*, *nad3*, *nad5*, and *nad6*). The incomplete codon T was used in genes including *cox2*, *nad3*, and *nad4*. Only *nad4L* used TAG as the termination codon (Supplementary Table S3). The two populations are identical in the usage of the initiation codon and termination codon.

**FIGURE 2:**
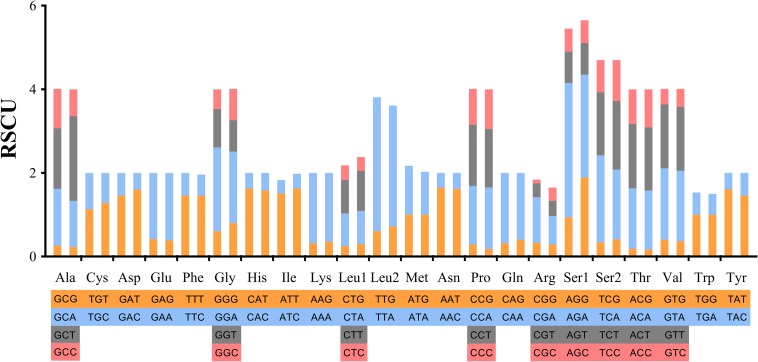
Relative synonymous codon usage in protein-coding genes of *Haemaphysalis longicornis* bisexual population and parthenogenetic population. The different amino acids are shown in four colors at most.

Codons including TTT (Phe), ATT (Ile) and AAA (Lys) showed the highest utilization rate. Codons of GCG (Ala), CCG (Pro), CGG (Arg), CGC (Arg) and ACG (Thr) containing “CG” showed relatively lower utilization rates, that is, less than 10 times (Supplementary Table S4). In tRNA, Phe and Leu2 were used more than 400 times in the bisexual population, and Phe, Ile and Lys were the most frequently used in the parthenogenetic population, being used more than 400 times. The lowest tRNA usage was Arg in the two reproductive populations (Figure 2).

### Transfer RNA and Ribosomal RNA

The mitochondrial genome in *H. longicornis* contains 22 tRNA and 2 rRNA. The secondary structure of all tRNA was successfully predicted, and the length ranged from 53 to 68 bp, with the longest *trnQ*, and the shortest *trnC* (Supplementary Figures S5, S6). The rRNA of *H. longicornis* displayed a significant AT bias. The total length of the tRNA in the two populations was identical, reaching 1,352 bp. The length of *rrnL* was 995 bp (AT = 82.21%), and the *rrnS* length was 778 bp (AT = 79.56%) in the bisexual population. In the parthenogenetic population, the *rrnL* length was 997 bp (AT = 82.25%), and the *rrnL* length was 777 bp (AT = 79.51%).

### Gene Rearrangement and Position Variance

**FIGURE 3:**
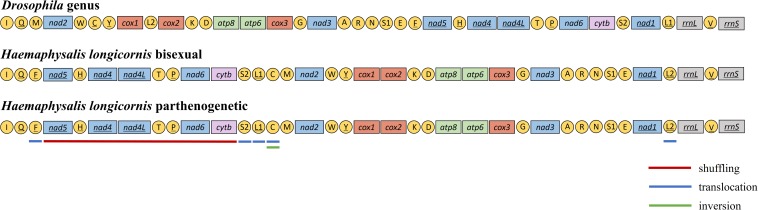
Mitochondrial gene arrangement characteristics of *Haemaphysalis longicornis*. The different color underlines highlighted the genes and rearrangements.

The mitochondrial genome of *H. longicornis* was characterized by obvious rearrangement in major genes. Five PCGs, including *nad5*-*nad4*-*nad4l*-*nad6*-*cytb*, were rearranged in new regions, which resulted in the displacement of the NCRs. The tRNA genes *trnF*, *trnH*, *trnT*, *trnP, trnS2*, *trnL1*, *trnC*, and *trnL2* changed the *Drosophila* genus arrangement. The displacement of tRNA was also observed in the two reproductive populations (Figure 3).

### Non-coding Regions and Gene Overlapping

Most mitochondrial genes of *H. longicornis* displayed overlapping regions or spacer regions. A total of 22 spacer regions and seven overlapping regions of genes were found in the mitochondrial genome of *H. longicornis*, with overlapping regions ranging from 1 to 9 bp. The special region was *atp6*: in the bisexual population, *atp6* overlapped by four bases, whereas in the parthenogenetic population, there was an interval region of eight bases. Four regions showed length differences between bisexual and parthenogenetic populations. The mitochondrial genome of *H. longicornis* contains three NCRs with a total length of more than 100 bp. NCR1 was located between *rrnL* and *trnV*, with a length of 159 bp; NCR2 was located between *rrnS* and *trnI*, with a length of 240 bp; and NCR3 was located between *trnL1* and *trnC*, with a length of 309 bp (Supplementary Table S3). The content of A+T in the NCRs of *H. longicornis* was only 60−70%.

### Phylogenetic and Homologous Gene Analysis

**FIGURE 4:**
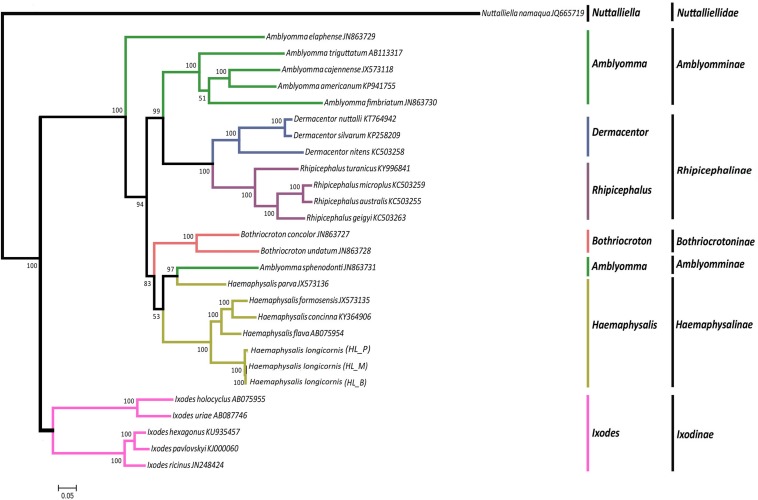
Phylogenetic tree combining 13 protein-coding genes of *Haemaphysalis longicornis*. Using ML analysis based on 24 Ixodidae mitochondrial genome data, different species were marked. The *Nuttalliella namaqua* (JQ665719) is the outgroup. In the phylogenetic tree, the scale bar represents the number of expected changes per site. The percentage of the ML bootstrap support was given at each node. The *Haemaphysalis longicornis* mitochondrial genome of the bisexual and parthenogenetic populations were in the same branch with strong support.

**FIGURE 5:**
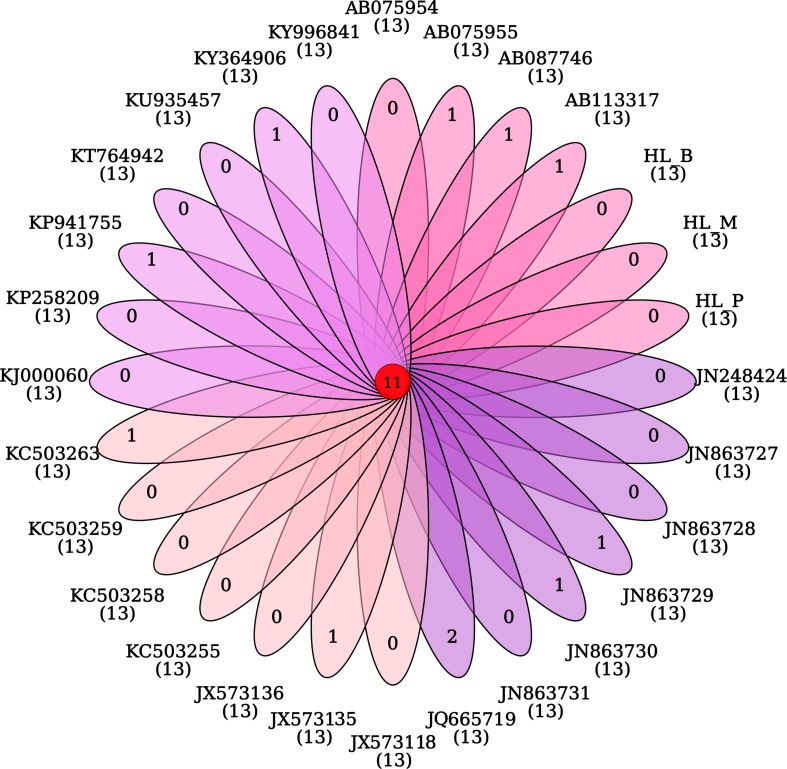
Venn map analysis of the homologous genes in *Haemaphysalis longicornis*. There are no unique homologous genes in the bisexual and parthenogenetic populations of the *Haemaphysalis longicornis* mitochondrial genome.

The phylogeny of the bisexual and the parthenogenetic population of *H. longicornis* was constructed with 24 tick species with complete mitochondrial genome available from the NCBI database. The results demonstrated that ticks in genera *Ixodes*, *Bothriocroton*, *Amblyomma* and *Haemaphysalis* were clustered into an independent branch, and ticks in *Dermacentor* and *Rhipicephalus* were grouped in Rhipicephalinae. *N. namaqua* and Ixodidae ticks were in different evolutionary branches. The two reproductive populations of *H. longicornis* were assigned to the cluster of the *Haemaphysalis* genus (Figure 4). In addition, homologous gene analysis showed that among 11 orthologous genes in these ticks, only one unique homologous gene was found in 10 ixodid ticks, and two novel homologous genes were found in *N. namaqua*. There were no unique homologous genes observed in the bisexual and parthenogenetic populations of *H. longicornis* (Figure 5).

### Quantitative Analysis of the Mitochondrial Protein-Coding Genes

The expression profiling of the 13 PCGs was detected in the females of the two populations during unfed, partially fed and engorgement, respectively. In the partially fed stage, the expression levels of most mitochondrial genes were similar to the unfed stage. The expression levels of *cox2*, *nad6*, *atp6*, and *atp8* genes were increased in the bisexual population, and *cytb* and *nad4* genes were significantly down regulated (*P* < 0.01). The expression of the *cox1* and *atp8* genes of the parthenogenetic population was significantly up regulated (*P* < 0.01). In contrast, the expression of the *nad3* genes was significantly decreased (*P* < 0.01). The *atp8* gene expression was considerably increased in the two populations at the partially fed and engorgement stages (Supplementary Figures S7, S8).

**FIGURE 6:**
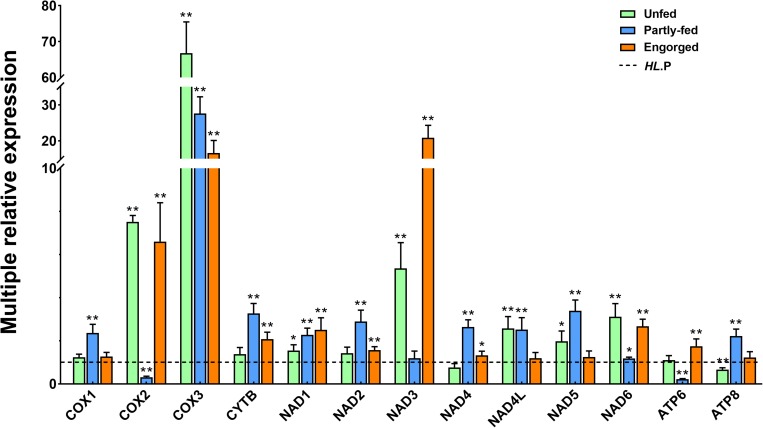
Comparative expression of the different feeding status of *Haemaphysalis longicornis*. The dotted line represents the expression of the parthenogenetic population. The asterisk indicates a level of significant difference (^∗^*P* < 0.05, ^∗∗^*P* < 0.01) in gene expression between the different groups.

When the gene expression of the parthenogenetic population was used as a reference, all genes in the bisexual population were differentially expressed, among which *cox2*, *cox3*, and *nad3* were most significantly expressed. The *cox3* gene expression of the bisexual population in the unfed stage was 60-fold upregulated, whereas it was 16-fold upregulated to the partially fed and engorgement stages. No changes were observed in the expression level of *nad3* in the unfed and engorgement stages in the parthenogenetic population, but it was significantly down regulated in the bisexual population (*P* < 0.01), which resulted in 20-fold variation between the two populations (Figure 6).

## Discussion

In the current study, the complete mitochondrial genomes of the bisexual and parthenogenetic populations of *H. longicornis* were assembled. The A+T content in *H. longicornis* was approximately 77%, and an obvious AT bias was also observed in most tick mitochondrial genomes (Black and Roehrdanz, 1998; Xiong et al., 2013; Williamsnewkirk et al., 2015). The A+T content affected both the codon usage pattern and amino acid composition of proteins (Breinholt and Kawahara, 2013). The GC and AT skew values of the two populations were negative, indicating that the mitochondrial genome has a slight preference in the usage of “T” and “C.” Previous studies have shown that the reversal of GC-skew might be related to the direction of replication but does not affect the direction of genes, whereas AT-skew can vary with changes in the direction of genes, replication and codon location (Wei et al., 2010). In the polymorphism analysis, we found that the mitochondrial sequences were entirely consistent between male and female ticks in the bisexual population, and no special structure was discovered, which maybe attributable to the maternal inheritance of the mitochondrial genome (Burger et al., 2013).

The arrangement of PCGs was similar to other tick species (Burger et al., 2013; Williamsnewkirk et al., 2015; Guo et al., 2016). The starting codon used by *H. longicornis* contained four common “ATN” types, and no special form of the first codon subtype was found. In *H. longicornis*, ATT was used as the initial codon for *cox1*, which is different from “CGA” used in some insect species (Zhu et al., 2013). Three termination codons, including TAA, TAG and “T,” were found in *H. longicornis*, and the incomplete termination codons “T” were common in mitochondrial genes (Pan et al., 2008; Dai et al., 2016), which might be converted to complete TAA codons by polyadenylation (Ojala et al., 1981).

In the tick *H. longicornis*, the most frequently used *trnS1* and *trnS2* displayed A and T bases preference in codon usage. Correspondingly, the TTA codon improved the higher usage of *trnL2*. A low utilization rate was observed in “CGC” and “CGG,” which resulted in the lower usage of *trnR*. In addition, *trnW* showed the lowest utilization rate. Changes in codon usage are generally regarded as important factors affecting protein expression levels (Zhou J. L. et al., 2004; Fang et al., 2015). Although some differences in codon usage were observed between the two reproductive populations, there was no significant difference in genetic structure or polymorphism between the bisexual and parthenogenetic populations.

The secondary structure of tRNA differs only at a few bases between the two reproductive populations. The DHU arms of *trnC* and *trnS1* were absent in the two populations, which were common features in ticks and insects (Jühling et al., 2009; Cameron, 2014; Williamsnewkirk et al., 2015; Yuan et al., 2015; Liu et al., 2018). The base mismatch of mitochondrial tRNA genes is a common phenomenon (Jühling et al., 2012), which mainly appears in four tRNA structures: the amino acid acceptor arm (AA), the TΨC arm (T), the anticodon arm (AC) and the dihydrouridine arm (DHU) (Zhang et al., 2012). A total of 11 base mismatches were observed in the tRNA genes of *H. longicornis*, among which G-U mismatches occurred 9 times, and the remaining two pairs were U-U mismatches. The mismatch was similar between the bisexual and parthenogenetic populations. The RNA editing process can correct the base mismatch without affecting the tRNA transport function (Bae et al., 2004). However, the base mismatch may jeopardize the survival ability of living organisms (Hendrickson, 2001) and hence result in the evolution of species (Watanabe et al., 1994). The gene length, base skew, and arrangement position of the rRNA in the bisexual and parthenogenetic *H. longicornis* were similar to those of other insects (Breinholt and Kawahara, 2013).

In most cases, the mitochondrial genomes displayed conservative gene arrangement and stable gene structure. Despite rapid evolution over time, the genomic arrangement of most arthropod mitochondria usually remains unchanged (Boore, 1999), and there is no similarity in mitochondrial gene rearrangement (Cameron et al., 2006). The genetic arrangement in the *Drosophila* genus was generally considered to be the original form of insects (Boore, 1999). Mitochondrial gene rearrangement includes major gene rearrangement and minor gene rearrangement (Boore and Brown, 2000). Like insects, two patterns of mitochondrial gene rearrangement were found in different genera of ticks. The minor rearrangement type was relatively common and occurred only in exchanges of the tRNA position (Cameron et al., 2007). This kind of rearrangement was common in *Ixodes* genus, but the rearrangement degree was significantly lower than that observed in other genera of ticks (Liu et al., 2018). The major gene rearrangement referred to the rearrangement or inversion of PCG or rRNA genes and infrequently occurred in insects. Liu et al. reported the major gene rearrangement from some tick genera, such as *Haemaphysalis*, *Amblyomma*, and *Rhipicephalus* (Liu et al., 2018). There were three types of tRNA gene position changes: shuffling (local rearrangements), translocation (cross-gene displacement) and inversion (change the encoding or transcriptional direction) (Dowton and Austin, 1999). Three different displacements were observed in *H. longicornis* tRNA: shuffling was found in *trnH*, *trnT* and *trnP*, translocation was observed in 5 genes (*trnF*, *trnS2*, *trnL1*, *trnC*, and *trnL2*), and *trnC* experienced gene inversion. The tRNA gene rearrangement was interpreted as a result of tandem duplication and random loss (Jühling et al., 2012; Xia et al., 2016), which could provide important genetic and phylogenetic information (Boore et al., 1998; Cameron et al., 2006).

Mitochondrial genomes generally have no introns. Although there are some intergenic regions, most of the arthropod mitochondrial genes are closely linked (Boore, 1999). The mitochondrial genomes of ticks contain different sizes of overlapping regions, including intergenic regions and NCRs. These regions are generally regarded as being involved in gene expression and regulation. The current study found three NCRs in the mitochondrial genome of *H. longicornis*. NCR1 was speculated to be a randomly insert fragment, which was also reported previously in *Dermacentor* and *Rhipicephalus* and possibly related to the tRNA structure (Cameron and Whiting, 2008; Burger et al., 2014b; Mccooke et al., 2015; Guo et al., 2016). The NCR2 and NCR3 were situated in similar positions with other tick species (Burger et al., 2012; Montagna et al., 2012; Williamsnewkirk et al., 2015). No difference was observed in the length or location of NCRs between the bisexual and parthenogenetic populations of *H. longicornis*. The NCRs were considered as a gene control region in arthropod, and the number of repeat sequences and conserved structures could directly affect the length and base skew of the NCRs (Simon, 1991; Montagna et al., 2012; Cameron, 2014; Li and Liang, 2018), which may be the reason for the lower A+T content of NCRs (60−70%) in *H. longicornis*. Recently, a “Tick-Box” structure was found in ticks, and metazoan, which is a 17-bp-long conserved sequence (*TTGyrTChwwwTwwGdA*) in the mitochondrial genome, was connected to transcription termination and gene alignment (Montagna et al., 2012). This conserved sequence was also found in two reproductive populations of *H. longicornis*, which were the same as other species of *Haemaphysalis* (*TTGCATCAATTTTTGGA*). Also, the conserved “ATGATAA” repeat sequence was found between *atp8* and *atp6* in *H. longicornis*, which has been reported in other ticks and insects (Stewart and Beckenbach, 2006; Williamsnewkirk et al., 2015).

Phylogenetic tree analysis showed that the independent branches of *Ixodes*, *Rhipicephalus*, *Amblyomma* and *Dermacentor* species were similar to the findings of Burger et al. (2014a). The conjoint analysis using all PCG genes could improve the stability and reliability of phylogenetic reconstruction (Sheffield et al., 2009). The two reproductive populations of *H. longicornis* were the most closely related and were less than the criteria for subspecies identification, which was consistent with previous results (Chen et al., 2010; Chen X. et al., 2012). Only one non-synonymous site of PCGs was observed in the two populations, whereas nearly 200 polymorphic sites were detected, but no apparent changes were observed in gene structure and arrangement under the evolutionary pressure of different environments.

The mitochondrion is essential for energy metabolism and is also involved in many critical processes, including cell transport, signal transduction, temperature regulation and immune activity (Brand, 2000; Detmer and Chan, 2007). Mitochondrial genes may lead to significant changes in metabolism and adaptability in the process of evolution (Ballard and Pichaud, 2014), and their genetic diversity was mainly formed by random genetic drift and natural selection (Sun et al., 2018). Multiple genes of *H. longicornis* were significantly upregulated in the partially fed and engorgement stages; among these genes, the most significant changes were detected in *atp6* and *atp8*, which are the core subunits of ATP synthase (Fontanillas et al., 2010). The expression of these genes might provide guarantees for oxidative phosphorylation (OXPHOS) of cells and changes in the metabolism of body temperature. In previous studies, *cox1* was considered to be the essential gene for COX activity changes (Uno et al., 2004; Singtripop et al., 2007). In the bisexual *H. longicornis*, the upregulation of *cox1* was not evident compared with the other two subunits (*cox2* and *cox3*). In parthenogenetic *H. longicornis*, changes in the *cox1* gene were noticeable, but down-regulation in *cox2* and *cox3* was observed. These results suggested that the dynamic changes of the *cox* subunit were not solely related to the *cox1* gene. The *cox3* gene in the bisexual population was considerably higher than that in the parthenogenetic population at each feeding status. This differential expression may be attributed to the living environment of the bisexual population, where it is characterized by higher altitude and latitude, and the climatic conditions of temperature and humidity are more severe.

The most specific gene in the NADH complex is *nad3* (Sanz et al., 2010). Only one non-synonymous site was observed in this gene, and this mutation was likely to change the gene expression patterns and functions in the two populations. The expression of *nad3* was significantly downregulated in the bisexual and parthenogenetic *H. longicornis* at different status, whereas it was also multiple times higher in the engorgement stage in parthenogenetic *H. longicornis* compared with that in the bisexual population. The structural changes of PCGs were required events for environmental adaptation, and most genes were maintained or upregulated during blood feeding or at the engorgement stage, which suggested that the metabolic level in the body was increased or the number of mitochondria was increased. These changes will guarantee rapid development and reproduction in *H. longicornis*.

## Conclusion

In the current study, the mitochondrial genomes of the bisexual and parthenogenetic *H. longicornis* were analyzed, and the gene structure and position arrangement were similar between the two reproductive populations. However, single nucleotide polymorphism analysis showed that approximately 200 bases were different. Phylogenetic analysis suggested that the bisexual and parthenogenetic populations were more closely related than the subspecies. Quantitative results of PCGs showed that the expression patterns of genes in the two reproductive populations were significantly distinctive at different feeding status, which may be similarly associated with environmental differences and reproductive patterns.

## Data Availability

The datasets generated for this study can be found in NCBI with the accession numbers: MK450606 (bisexual populations of *H. longicornis*) and MK439888 (parthenogenetic populations of *H. longicornis*).

## Ethics Statement

All experimental procedures were approved by the Animal Ethics Committee of Hebei Normal University (protocol number: IACUC-157026).

## Author Contributions

ZY conceived the experiments. TW performed the experiments. SZ and TP analyzed the data. ZY and TW drafted and edited the manuscript. JL reviewed and corrected the manuscript.

## Conflict of Interest Statement

The authors declare that the research was conducted in the absence of any commercial or financial relationships that could be construed as a potential conflict of interest.

## Footnotes

1 https://sourceforge.net/projects/soapdenovo2/

2 http://ogdraw.mpimp-golm.mpg.de/

3 http://mitos.bioinf.uni-leipzig.de/index.py

4 http://www.geneontology.org

5 http://lowelab.ucsc.edu/tRNAscan-SE

6 http://tandem.bu.edu/trf/trf.html

7 http://bio-bwa.sourceforge.net

8 https://software.broadinstitute.org/gatk

9 http://orthomcl.org/orthomcl

10 https://www.ncbi.nlm.nih.gov/

11 https://cme.h-its.org/exelixis/web/software/raxml/index.html

12 https://mafft.cbrc.jp/alignment/software/

13 http://molevol.cmima.csic.es/castresana/Gblocks.html

## Supplementary Material

FIGURE S1 Mitochondrial genome circular map of *Haemaphysalis longicornis* bisexual female.

FIGURE S2 Mitochondrial genome circular map of *Haemaphysalis longicornis* bisexual male.

FIGURE S3 Mitochondrial genome circular map of the *Haemaphysalis longicornis* parthenogenetic population.

FIGURE S4 Nucleotide polymorphisms in the parthenogenetic population with the bisexual population considered as the reference.

FIGURE S5 Secondary structures of tRNA in the bisexual population of *Haemaphysalis longicornis*.

FIGURE S6 Secondary structures of tRNAs in the parthenogenetic population of *Haemaphysalis longicornis*.

FIGURE S7 Quantitative expression of the different feeding status of the bisexual population. The asterisk indicates a level of significant difference (^∗^*P* < 0.05, ^∗∗^*P* < 0.01) in gene expression between the different groups.

FIGURE S8 Quantitative expression of the different feeding status of the parthenogenetic population. The asterisk indicates a level of significant difference (^∗^*P* < 0.05, ^∗∗^*P* < 0.01) in gene expression between the different groups.

TABLE S1 Original mitochondrial genome sequencing data of *Haemaphysalis longicornis*.

TABLE S2 Quantitative primers in the mitochondrial genome of *Haemaphysalis longicornis*.

TABLE S3 Comparison of the mitochondrial gene position and arrangement in the bisexual and parthenogenetic population of *Haemaphysalis longicornis*.

TABLE S4 Codon usage of *Haemaphysalis longicornis* in the mitochondrial protein-coding genes.
