# Supplementary figures and images for: The Complete Mitochondrial Genome and Expression Profile of Mitochondrial Protein-Coding Genes in the Bisexual and Parthenogenetic Haemaphysalis longicornis
Source: Front Physiol. 2019 Jul 30;10:982. doi: 10.3389/fphys.2019.00982 (PMC6682753; doi:10.3389/fphys.2019.00982)

## Slide 1
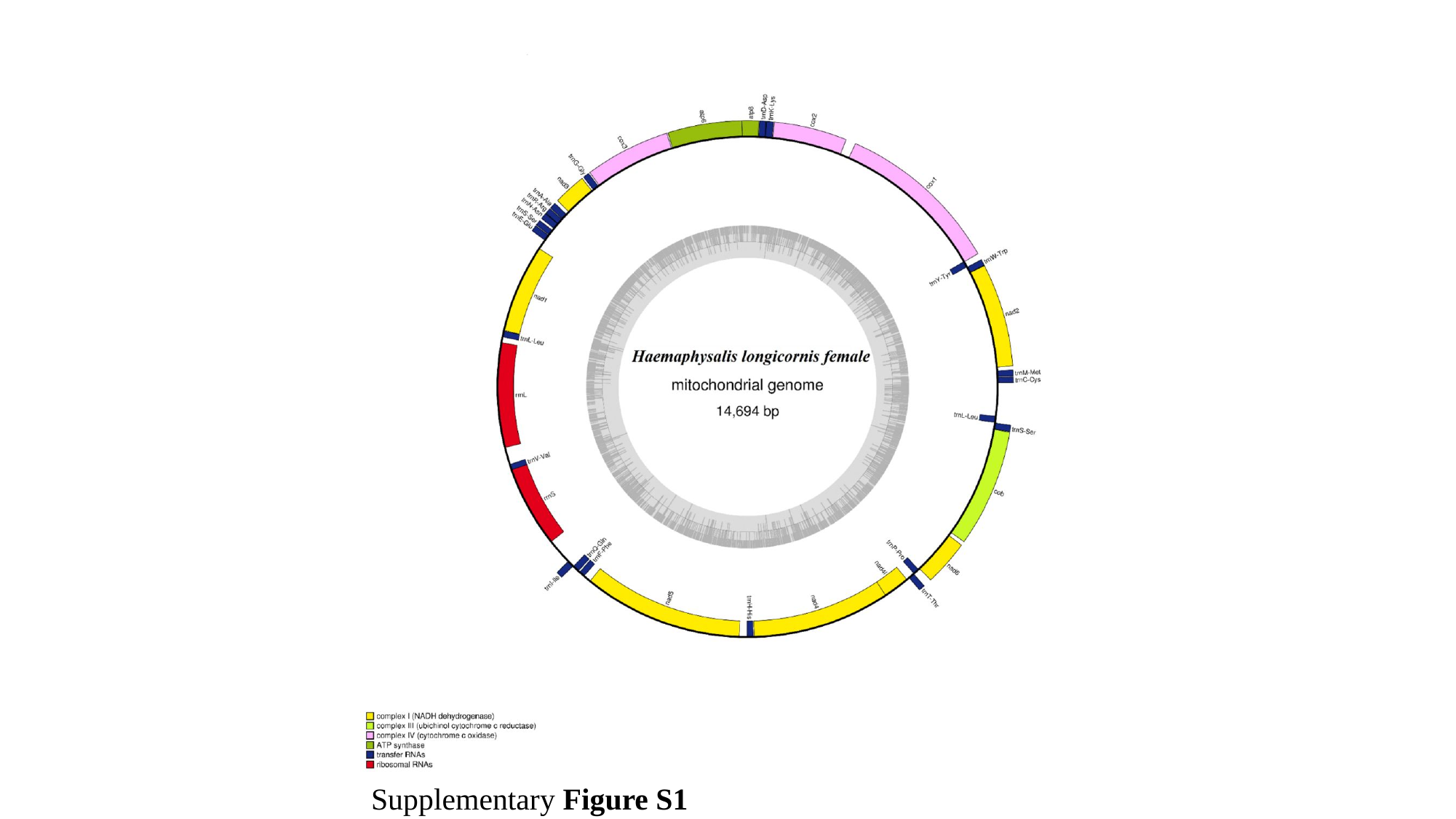

Supplementary Figure S1

Supplement: FIGURE S1 — Mitochondrial genome circular map of Haemaphysalis longicornis bisexual female. [file Presentation_1.PPTX]

## Slide 1
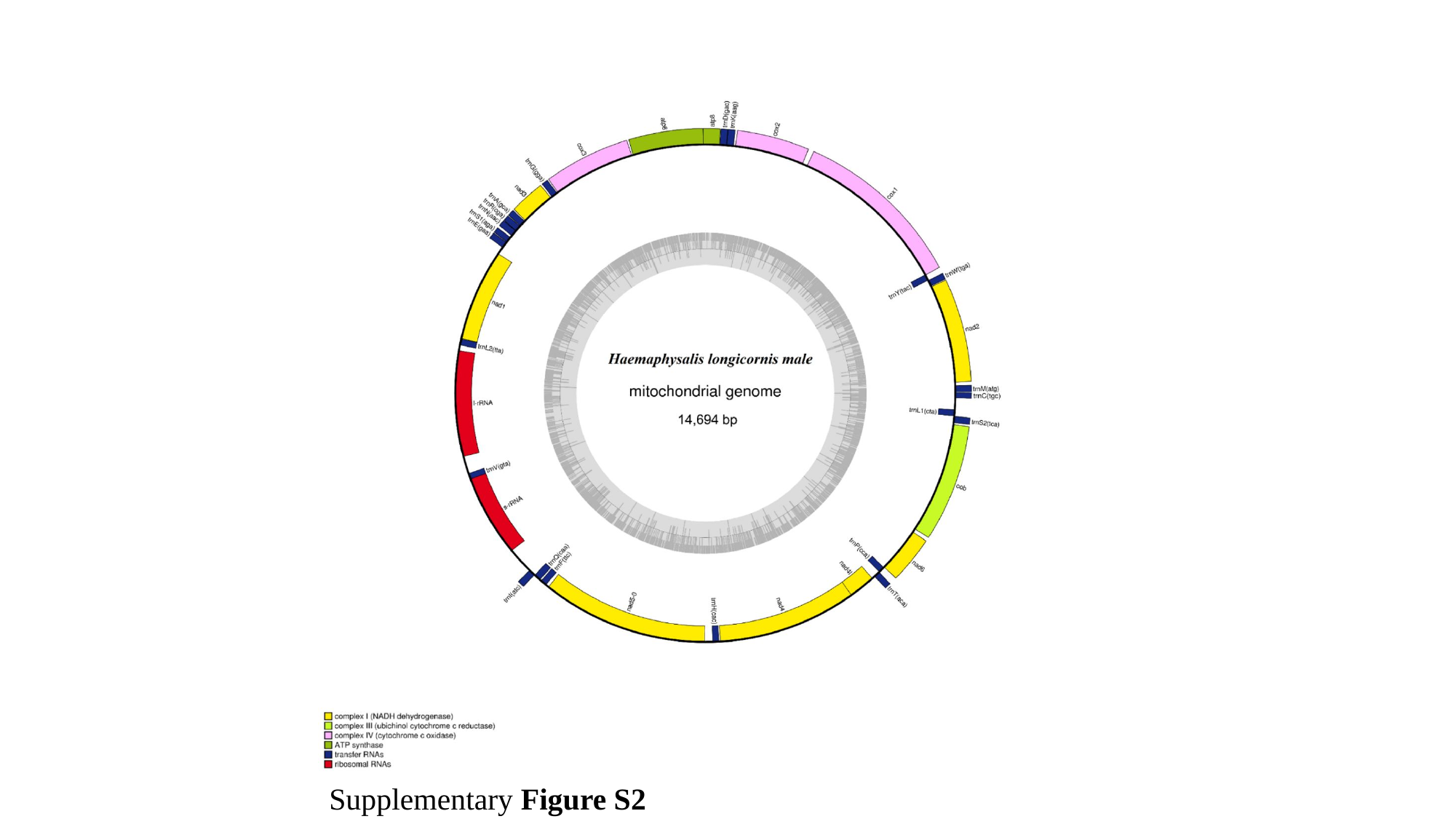

Supplementary Figure S2

Supplement: FIGURE S2 — Mitochondrial genome circular map of Haemaphysalis longicornis bisexual male. [file Presentation_2.PPTX]

## Slide 1
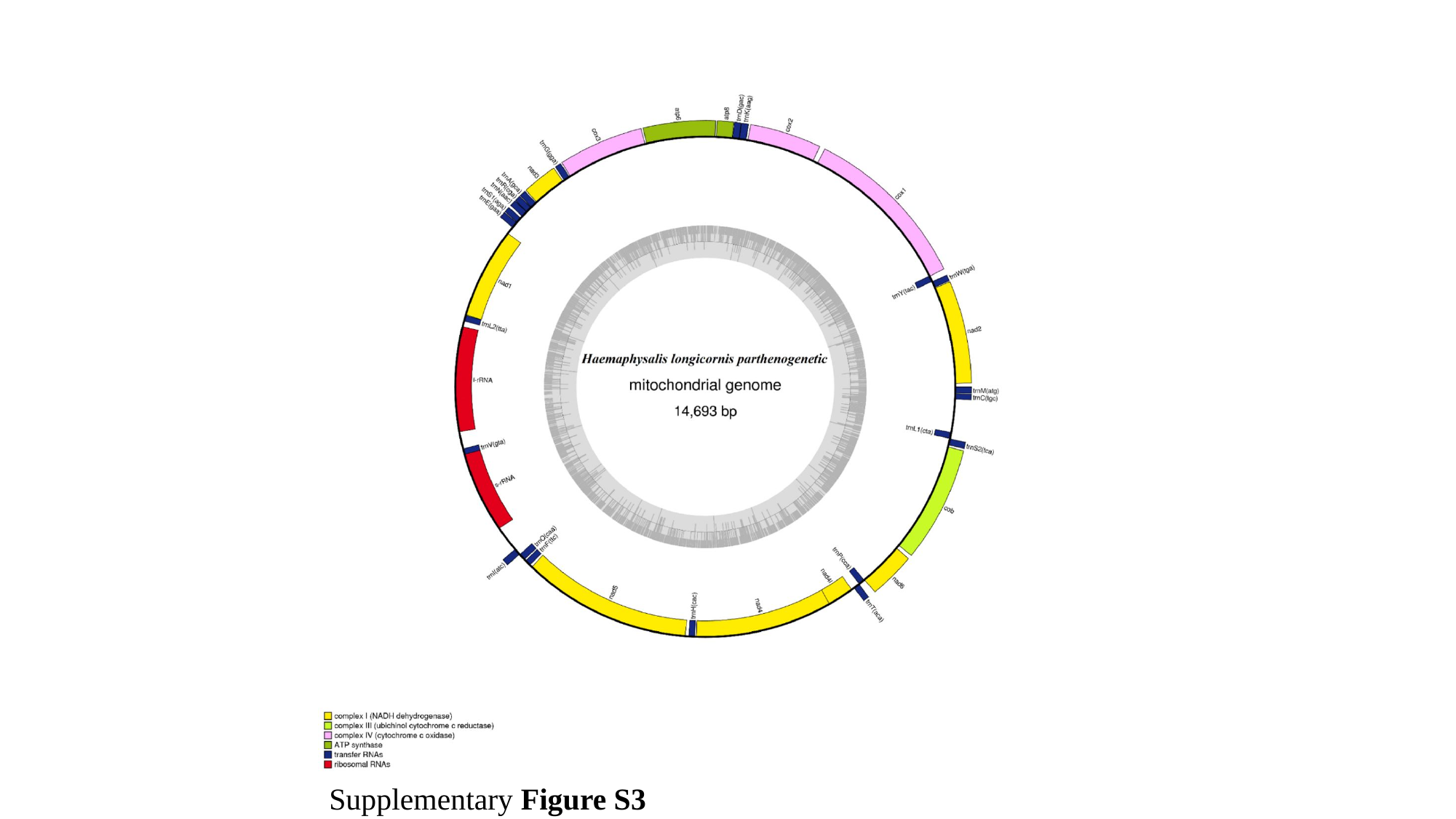

Supplementary Figure S3

Supplement: FIGURE S3 — Mitochondrial genome circular map of the Haemaphysalis longicornis parthenogenetic population. [file Presentation_3.PPTX]

## Slide 1
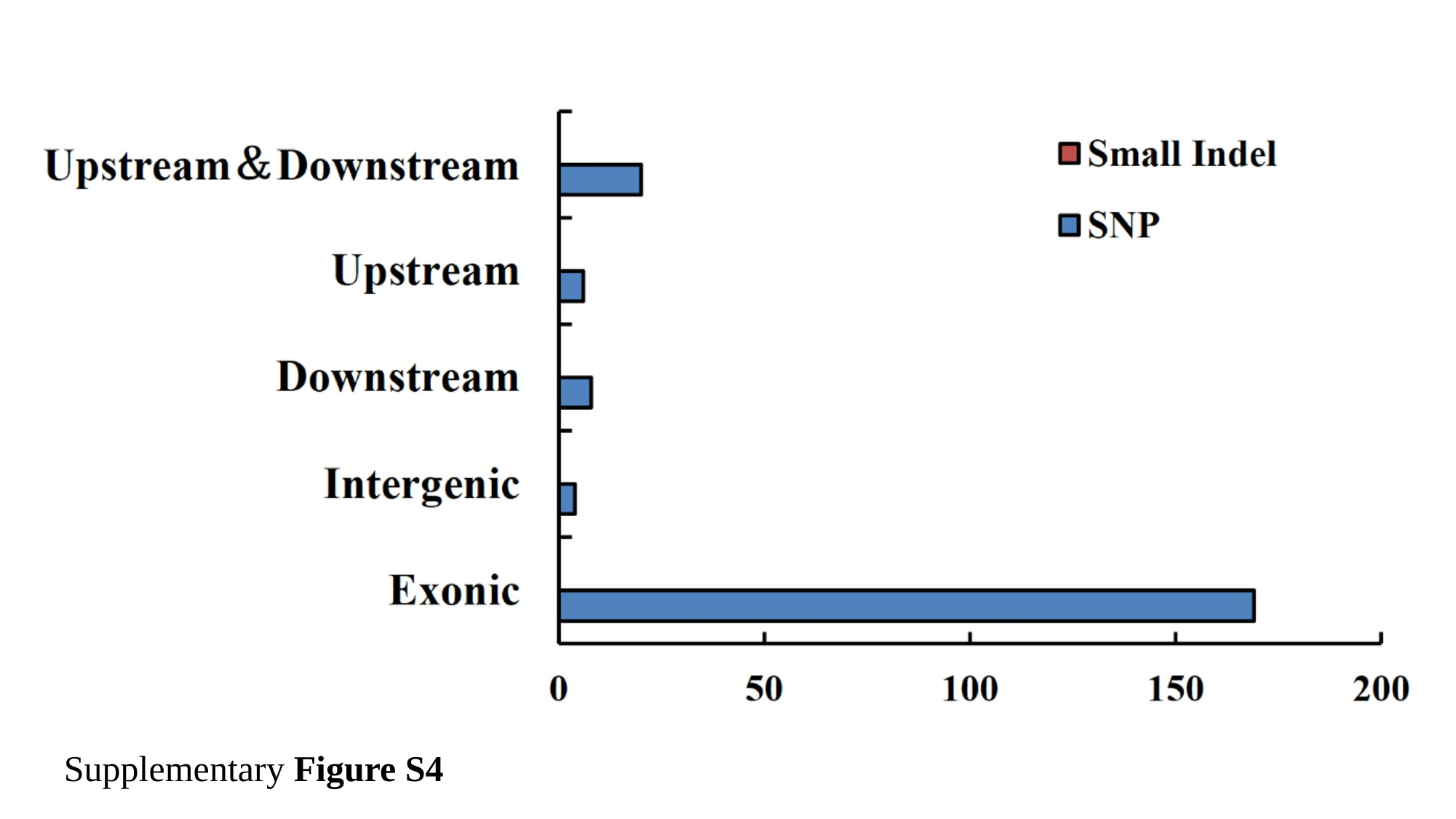

Supplementary Figure S4

Supplement: FIGURE S4 — Nucleotide polymorphisms in the parthenogenetic population with the bisexual population considered as the reference. [file Presentation_4.PPTX]

## Slide 1
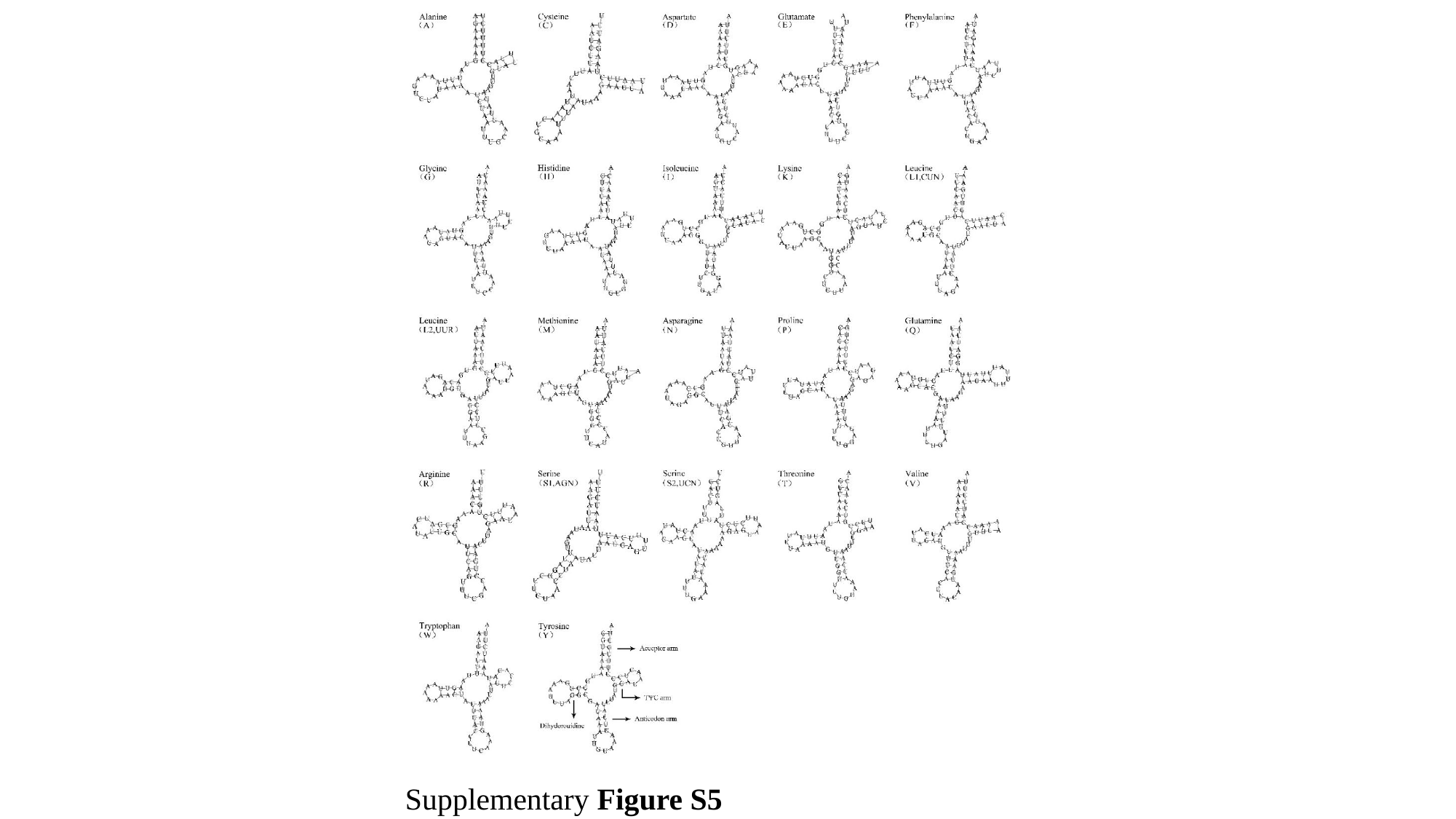

Supplementary Figure S5

Supplement: FIGURE S5 — Secondary structures of tRNA in the bisexual population of Haemaphysalis longicornis. [file Presentation_5.PPTX]

## Slide 1
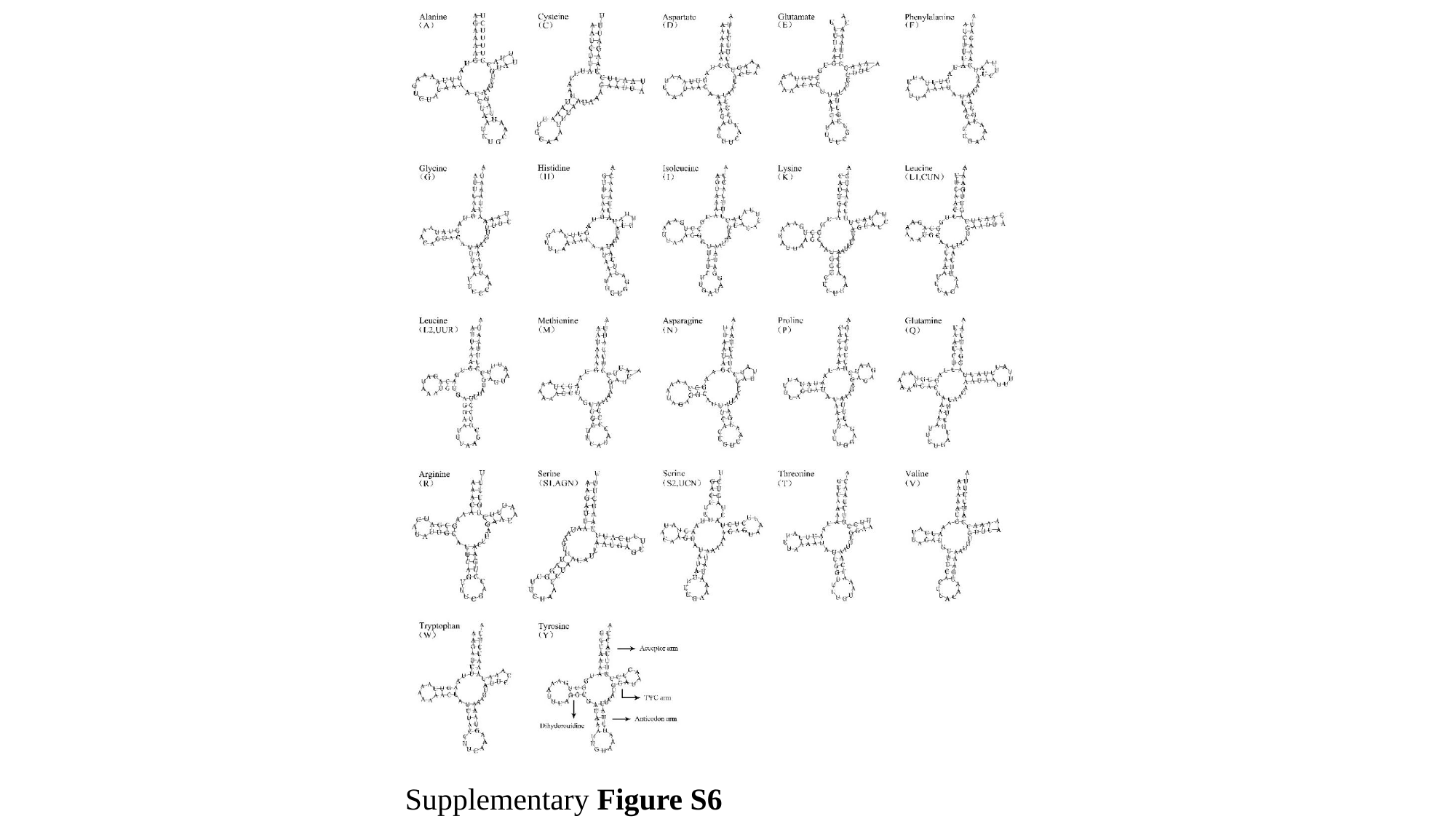

Supplementary Figure S6

Supplement: FIGURE S6 — Secondary structures of tRNAs in the parthenogenetic population of Haemaphysalis longicornis. [file Presentation_6.PPTX]

## Slide 1
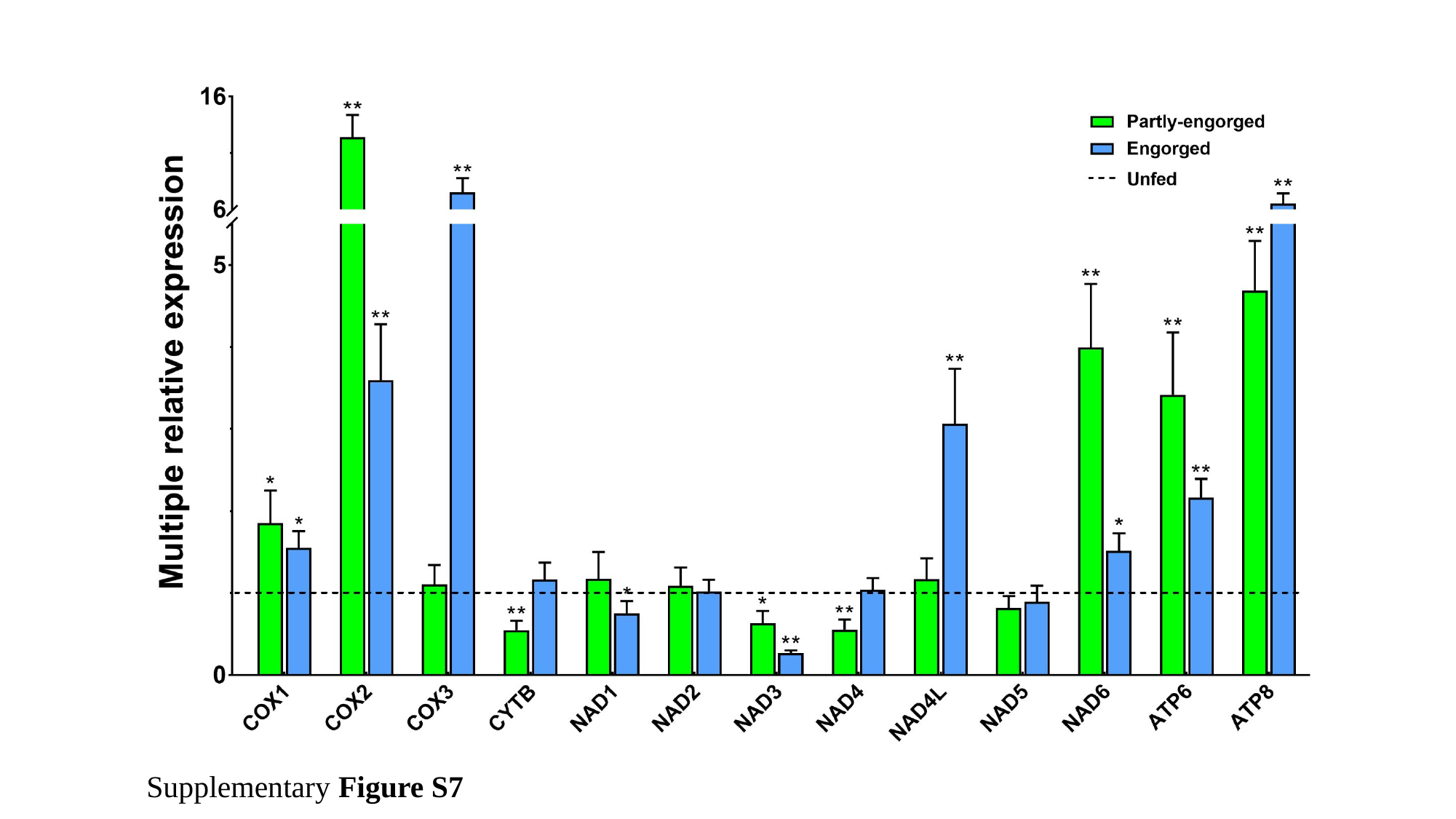

Supplementary Figure S7

Supplement: FIGURE S7 — Quantitative expression of the different feeding status of the bisexual population. The asterisk indicates a level of significant difference (∗P < 0.05, ∗∗P < 0.01) in gene expression between the different groups. [file Presentation_7.PPTX]

## Slide 1
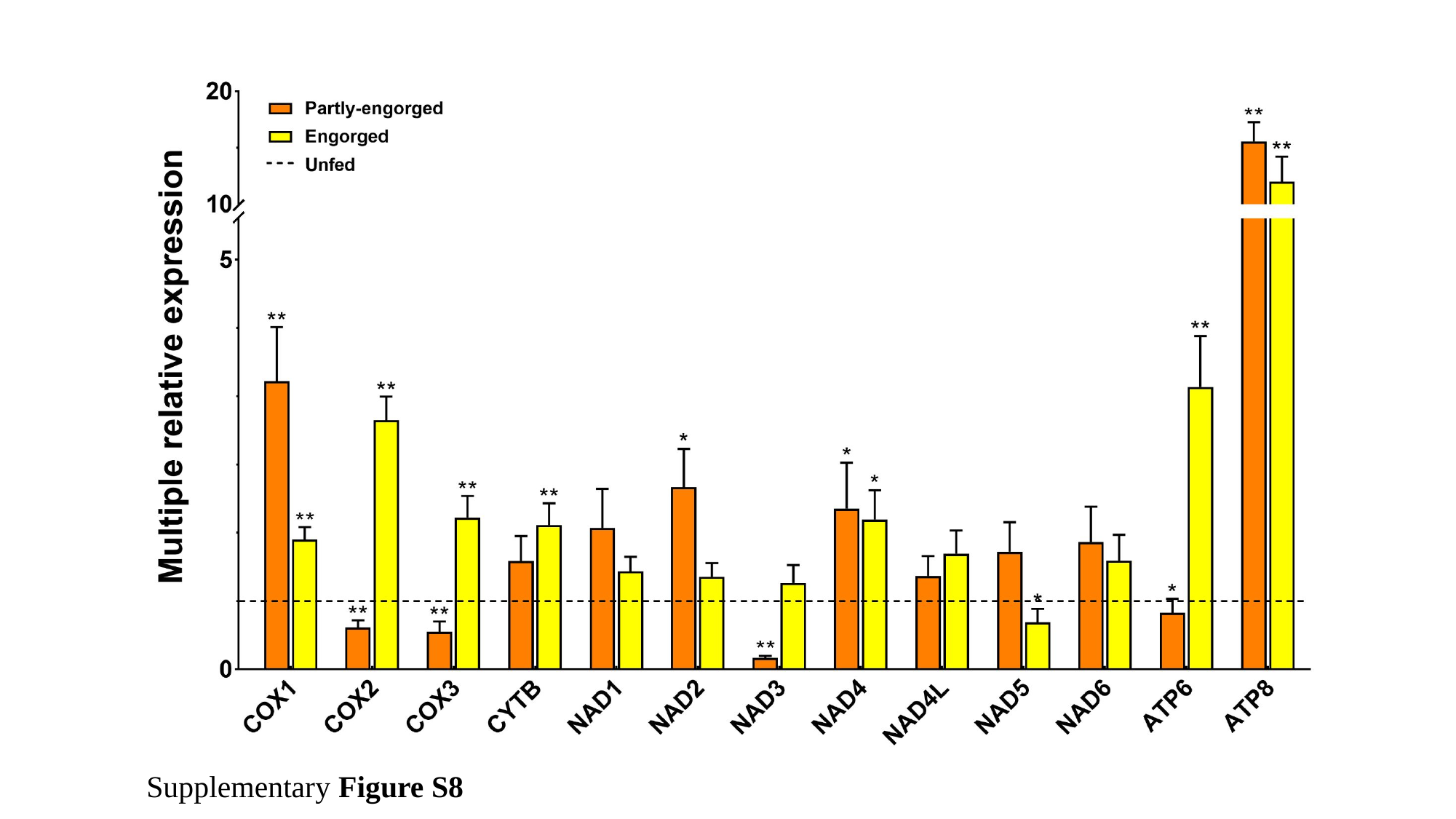

Supplementary Figure S8

Supplement: FIGURE S8 — Quantitative expression of the different feeding status of the parthenogenetic population. The asterisk indicates a level of significant difference (∗P < 0.05, ∗∗P < 0.01) in gene expression between the different groups. [file Presentation_8.PPTX]
