# Supplementary material for: The Complete Mitochondrial Genome and Expression Profile of Mitochondrial Protein-Coding Genes in the Bisexual and Parthenogenetic Haemaphysalis longicornis
Source: Front Physiol. 2019 Jul 30;10:982. doi: 10.3389/fphys.2019.00982 (PMC6682753; doi:10.3389/fphys.2019.00982)
Supplement: TABLE S1 — Original mitochondrial genome sequencing data of Haemaphysalis longicornis. [file Table_1.DOCX]

| Sample | Read Length (bp) | Raw Reads (bp) | Clean reads (bp) | Q20% | Scaffold N50(bp) | Largest length (bp) |
| --- | --- | --- | --- | --- | --- | --- |
| HL_B | 150 | 6292409486 | 5774584822 | 95.21 | 14694 | 14694 |
| HL_M | 150 | 6089707992 | 5582567668 | 95.2 | 14694 | 14694 |
| HL_P | 150 | 5896039318 | 5442297788 | 95.44 | 14693 | 14693 |

Supplementary **Table S1** The mitochondrial genome original sequencing data of *H. longicornis*.
