# Supplementary material for: The Complete Mitochondrial Genome and Expression Profile of Mitochondrial Protein-Coding Genes in the Bisexual and Parthenogenetic Haemaphysalis longicornis
Source: Front Physiol. 2019 Jul 30;10:982. doi: 10.3389/fphys.2019.00982 (PMC6682753; doi:10.3389/fphys.2019.00982)
Supplement: TABLE S2 — Quantitative primers in the mitochondrial genome of Haemaphysalis longicornis. [file Table_2.DOCX]

Supplementary **Table S2** Quantitative primers in the mitochondrial genome of *H. longicornis*.

| Gene | Size (bp) | | Primer sequence (5'–3') | Products (bp) |
| --- | --- | --- | --- | --- |
| *Cox1* | F | 18 | TTAGCAGGAGCCTCATCA | 145 |
|  | R | 21 | GTAAGGATAGCAGAAGGAGAA |  |
| *Cox2* | F | 21 | CCACTCATGATCACTTCCCTC | 132 |
|  | R | 21 | TGGTTTGCTCCGCAAATTTCA |  |
| *Cox3* | F | 22 | CAGGAATCTCTGTTACTTGAAG | 197 |
|  | R | 21 | CCCATGAAATCCAGTAGTTAA |  |
| *Cytb* | F | 20 | CTCAAGAGACATTTCAACTG | 144 |
|  | R | 20 | TAGATACCACGAGCAATATG |  |
| *Nad1* | F | 20 | TTCTGATTCTCCTTCTGCTA | 219 |
|  | R | 18 | CGAGGGTTTGCTCAAGTA |  |
| *Nad2* | F | 20 | AATATCTCTTGGCGGAATAC | 166 |
|  | R | 20 | GAAAGTTACTAAAGCTGGAG |  |
| *Nad3* | F | 25 | TGTAATTATTATCCCATTTCCAATC | 128 |
|  | R | 25 | ACCAATCAATTATTCCTAATTTTCA |  |
| *Nad4* | F | 23 | AATAATAACGACCCATAAGAACC | 164 |
|  | R | 22 | TTAATAATTTTGTCCCTTTGAG |  |
| *NadL* | F | 20 | GGAATTGAGCCAAGTACATA | 153 |
|  | R | 20 | GTATGTGAAGCCTCTATTGG |  |
| *Nad5* | F | 18 | CTGCGGTCACTAATGTTG | 227 |
|  | R | 20 | TCGAGTAGGAGATGTAATGA |  |
| *Nad6* | F | 29 | ATTACTAACATTATATATATCTTTAGCTT | 138 |
|  | R | 23 | TTATTATTAGGGCTTAGTCTAAC |  |
| *Atp6* | F | 20 | ACGTTTTTACCGCATCAAGTCA | 135 |
|  | R | 20 | TCGGAGAGCCTAATGGAACT |  |
| *Atp8* | F | 18 | CCACAAATTTTTCCAATGAATTG | 153 |
|  | R | 26 | TTATCATTTAAATAGTAAATTGTTAA |  |
| *Actin* | F | 20 | AGCGTGGTATCCTCACTCTG | 189 |
|  | R | 18 | ACATGATCTGCGTCATCTTCTC |  |
