# Supplementary material for: The Complete Mitochondrial Genome and Expression Profile of Mitochondrial Protein-Coding Genes in the Bisexual and Parthenogenetic Haemaphysalis longicornis
Source: Front Physiol. 2019 Jul 30;10:982. doi: 10.3389/fphys.2019.00982 (PMC6682753; doi:10.3389/fphys.2019.00982)
Supplement: TABLE S3 — Comparison of the mitochondrial gene position and arrangement in the bisexual and parthenogenetic population of Haemaphysalis longicornis. [file Table_3.DOCX]

Supplementary **Table S3** Comparative of the mitochondrial gene position and arrangement in the bisexual andparthenogenetic population of *H. longicornis*.

| Genes | HL.B | |  | HL.P | |  | HL.B / HL.P | | | | | |
| --- | --- | --- | --- | --- | --- | --- | --- | --- | --- | --- | --- | --- |
|  | Start/stop Position | |  | Start/stop Position | |  | Intergenic | Length | Strand | Anticodon | Start | Stop |
| *trnM* | 1 | 61 |  | 1 | 61 |  | 0 | 61 | + | ATG |  |  |
| *nad2* | 92 | 1012 |  | 92 | 1012 |  | 30 | 921 | + |  | ATT | TAA |
| *trnW* | 1017 | 1077 |  | 1017 | 1077 |  | 4 | 61 | + | TGA |  |  |
| *trnY* | 1076 | 1138 |  | 1076 | 1138 |  | -2 | 63 | - | TAC |  |  |
| *cox1* | 1131 | 2648 |  | 1131 | 2648 |  | -8 | 1518 | + |  | ATT | TAA |
| *cox2* | 2692 | 3336 |  | 2692 | 3336 |  | 43 | 645 | + |  | ATT | T |
| *trnK* | 3351 | 3417 |  | 3351 | 3417 |  | 14 | 67 | + | AAG |  |  |
| *trnD* | 3417 | 3479 |  | 3417 | 3479 |  | -1 | 63 | + | GAC |  |  |
| *atp8* | 3481 | 3633 |  | 3481 | 3633 |  | 1 | 153 | + |  | ATA | TAA |
| *atp6* | 3630 | 4289 |  | 3642 | 4289 |  | -4/8 | 660/648 | + |  | ATG | TAA |
| *cox3* | 4302 | 5069 |  | 4302 | 5069 |  | 12 | 768 | + |  | ATA | TAA |
| *trnG* | 5078 | 5137 |  | 5078 | 5137 |  | 8 | 60 | + | GGA |  |  |
| *nad3* | 5150 | 5467 |  | 5150 | 5467 |  | 12 | 318 | + |  | ATT | T |
| *trnA* | 5475 | 5536 |  | 5475 | 5536 |  | 7 | 62 | + | GCA |  |  |
| *trnR* | 5539 | 5596 |  | 5539 | 5596 |  | 2 | 58 | + | CGA |  |  |
| *trnN* | 5596 | 5656 |  | 5596 | 5656 |  | -1 | 61 | + | AAC |  |  |
| *trnS1* | 5675 | 5730 |  | 5675 | 5730 |  | 18 | 56 | + | AGA |  |  |
| *trnE* | 5733 | 5793 |  | 5733 | 5793 |  | 2 | 61 | + | GAA |  |  |
| *nad1* | 5860 | 6726 |  | 5860 | 6726 |  | 66 | 867 | - |  | ATT | TAA |
| *trnL2* | 6727 | 6788 |  | 6727 | 6787 |  | 0 | 62/61 | - | TTA |  |  |
| *rrnL* | 6837 | 7831 |  | 6836 | 7832 |  | 48 | 995/997 | - |  |  |  |
| NCR1 | 7832 | 7990 |  | 7833 | 7991 |  |  | 159 |  |  |  |  |
| *trnV* | 7991 | 8049 |  | 7992 | 8050 |  | 0 | 59 | - | GTA |  |  |
| *rrnS* | 8041 | 8818 |  | 8042 | 8817 |  | -9 | 778/776 | - |  |  |  |
| NCR2 | 8819 | 9058 |  | 8818 | 9057 |  |  | 240 |  |  |  |  |
| *trnI* | 9059 | 9124 |  | 9058 | 9123 |  | 0 | 66 | + | ATC |  |  |
| *trnQ* | 9125 | 9192 |  | 9124 | 9191 |  | 0 | 68 | - | CAA |  |  |
| *trnF* | 9204 | 9265 |  | 9203 | 9264 |  | 11 | 62 | - | TTC |  |  |
| *nad5* | 9292 | 10854 |  | 9285 | 10895 |  | 26/20 | 1563/1611 | - |  | ATT | TAA |
| *trnH* | 10924 | 10984 |  | 10923 | 10983 |  | 69/27 | 61 | - | CAC |  |  |
| *nad4* | 10995 | 12299 |  | 10994 | 12298 |  | 10 | 1305 | - |  | ATG | T |
| *nad4l* | 12296 | 12547 |  | 12295 | 12546 |  | -4 | 252 | - |  | ATT | TAG |
| *trnT* | 12571 | 12630 |  | 12570 | 12629 |  | 23 | 60 | + | ACA |  |  |
| *trnP* | 12631 | 12692 |  | 12630 | 12691 |  | 0 | 62 | - | CCA |  |  |
| *nad6* | 12695 | 13117 |  | 12694 | 13116 |  | 2 | 423 | + |  | ATC | TAA |
| *cob* | 13149 | 14189 |  | 13148 | 14182 |  | 31 | 1041/1035 | + |  | ATA | TAA |
| *trnS2* | 14205 | 14267 |  | 14204 | 14266 |  | 15/21 | 63 | + | TCA |  |  |
| *trnL1* | 14267 | 14329 |  | 14266 | 14328 |  | -1 | 63 | - | CTA |  |  |
| NCR3 | 14330 | 14638 |  | 14329 | 14637 |  |  | 309 |  |  |  |  |
| *trnC* | 14639 | 14691 |  | 14638 | 14690 |  | 3 | 53 | + | TGC |  |  |
