# Supplementary material for: The Complete Mitochondrial Genome and Expression Profile of Mitochondrial Protein-Coding Genes in the Bisexual and Parthenogenetic Haemaphysalis longicornis
Source: Front Physiol. 2019 Jul 30;10:982. doi: 10.3389/fphys.2019.00982 (PMC6682753; doi:10.3389/fphys.2019.00982)
Supplement: TABLE S4 — Codon usage of Haemaphysalis longicornis in the mitochondrial protein-coding genes. [file Table_4.DOCX]

Supplementary **Table S4** Codon usage of *H. longicornis* in the mitochondrial protein-coding genes.

| AmAcid | Codon | HL.B | | |  | HL.P | | |
| --- | --- | --- | --- | --- | --- | --- | --- | --- |
|  |  | /1000 | Number | Fraction |  | /1000 | Number | Fraction |
| Ala | GCG | 1.22 | 6 | 0.06 |  | 0.82 | 4 | 0.06 |
|  | GCA | 6.53 | 32 | 0.34 |  | 3.88 | 19 | 0.28 |
|  | GCT | 6.94 | 34 | 0.36 |  | 7.15 | 35 | 0.51 |
|  | GCC | 4.49 | 22 | 0.23 |  | 2.25 | 11 | 0.16 |
|  |  |  |  |  |  |  |  |  |
| Cys | TGT | 6.12 | 30 | 0.57 |  | 10.62 | 52 | 0.64 |
|  | TGC | 4.70 | 23 | 0.43 |  | 5.92 | 29 | 0.36 |
|  |  |  |  |  |  |  |  |  |
| Asp | GAT | 11.02 | 54 | 0.73 |  | 12.25 | 60 | 0.80 |
|  | GAC | 4.08 | 20 | 0.27 |  | 3.06 | 15 | 0.20 |
|  |  |  |  |  |  |  |  |  |
| Glu | GAG | 4.49 | 22 | 0.21 |  | 3.27 | 16 | 0.19 |
|  | GAA | 16.95 | 83 | 0.79 |  | 13.68 | 67 | 0.81 |
|  |  |  |  |  |  |  |  |  |
| Phe | TTT | 70.44 | 345 | 0.73 |  | 71.47 | 350 | 0.73 |
|  | TTC | 25.72 | 126 | 0.27 |  | 26.34 | 129 | 0.27 |
|  |  |  |  |  |  |  |  |  |
| Gly | GGG | 3.67 | 18 | 0.15 |  | 3.06 | 15 | 0.20 |
|  | GGA | 12.05 | 59 | 0.50 |  | 6.53 | 32 | 0.43 |
|  | GGT | 5.51 | 27 | 0.23 |  | 2.86 | 14 | 0.19 |
|  | GGC | 2.86 | 14 | 0.12 |  | 2.86 | 14 | 0.19 |
|  |  |  |  |  |  |  |  |  |
| His | CAT | 17.97 | 88 | 0.81 |  | 15.32 | 75 | 0.79 |
|  | CAC | 4.08 | 20 | 0.19 |  | 4.08 | 20 | 0.21 |
|  |  |  |  |  |  |  |  |  |
| Ile | ATT | 65.13 | 319 | 0.82 |  | 74.13 | 363 | 0.82 |
|  | ATC | 13.88 | 68 | 0.18 |  | 15.93 | 78 | 0.18 |
|  |  |  |  |  |  |  |  |  |
| Lys | AAG | 12.66 | 62 | 0.16 |  | 16.13 | 79 | 0.18 |
|  | AAA | 66.97 | 328 | 0.84 |  | 73.51 | 360 | 0.82 |
|  |  |  |  |  |  |  |  |  |
| Leu1 | CTG | 6.12 | 30 | 0.04 |  | 6.13 | 30 | 0.05 |
|  | CTA | 18.99 | 93 | 0.13 |  | 15.93 | 78 | 0.13 |
|  | CTT | 19.60 | 96 | 0.13 |  | 19.40 | 95 | 0.16 |
|  | CTC | 8.57 | 42 | 0.06 |  | 6.74 | 33 | 0.06 |
|  |  |  |  |  |  |  |  |  |
| Leu2 | TTG | 14.90 | 73 | 0.10 |  | 14.50 | 71 | 0.12 |
|  | TTA | 77.99 | 382 | 0.53 |  | 58.40 | 286 | 0.48 |
|  |  |  |  |  |  |  |  |  |
| Met | ATG | 10.62 | 52 | 0.17 |  | 10.82 | 53 | 0.19 |
|  | ATA | 50.22 | 246 | 0.83 |  | 46.76 | 229 | 0.81 |
|  |  |  |  |  |  |  |  |  |
| Asn | AAT | 57.37 | 281 | 0.83 |  | 65.35 | 320 | 0.81 |
|  | AAC | 12.05 | 59 | 0.17 |  | 15.11 | 74 | 0.19 |
|  |  |  |  |  |  |  |  |  |
| Pro | CCG | 1.84 | 9 | 0.07 |  | 1.02 | 5 | 0.04 |
|  | CCA | 8.98 | 44 | 0.35 |  | 8.58 | 42 | 0.37 |
|  | CCT | 9.39 | 46 | 0.37 |  | 8.17 | 40 | 0.35 |
|  | CCC | 5.51 | 27 | 0.21 |  | 5.51 | 27 | 0.24 |
|  |  |  |  |  |  |  |  |  |
| Gln | CAG | 4.08 | 20 | 0.16 |  | 4.70 | 23 | 0.20 |
|  | CAA | 21.64 | 106 | 0.84 |  | 18.58 | 91 | 0.80 |
|  |  |  |  |  |  |  |  |  |
| Arg | CGG | 1.43 | 7 | 0.18 |  | 1.63 | 8 | 0.17 |
|  | CGA | 4.70 | 23 | 0.59 |  | 3.88 | 19 | 0.41 |
|  | CGT | 1.43 | 7 | 0.18 |  | 2.04 | 10 | 0.22 |
|  | CGC | 0.41 | 2 | 0.05 |  | 1.84 | 9 | 0.20 |
|  |  |  |  |  |  |  |  |  |
| Ser1 | AGG | 4.08 | 20 | 0.05 |  | 10.82 | 53 | 0.11 |
|  | AGA | 13.88 | 68 | 0.17 |  | 14.09 | 69 | 0.14 |
|  | AGT | 8.17 | 40 | 0.10 |  | 9.19 | 45 | 0.09 |
|  | AGC | 5.92 | 29 | 0.07 |  | 6.53 | 32 | 0.07 |
|  |  |  |  |  |  |  |  |  |
| Ser2 | TCG | 3.67 | 18 | 0.04 |  | 4.90 | 24 | 0.05 |
|  | TCA | 22.46 | 110 | 0.27 |  | 20.22 | 99 | 0.21 |
|  | TCT | 16.33 | 80 | 0.20 |  | 19.81 | 97 | 0.20 |
|  | TCC | 8.37 | 41 | 0.10 |  | 11.84 | 58 | 0.12 |
|  |  |  |  |  |  |  |  |  |
| Thr | ACG | 1.84 | 9 | 0.05 |  | 1.63 | 8 | 0.04 |
|  | ACA | 14.09 | 69 | 0.36 |  | 13.27 | 65 | 0.35 |
|  | ACT | 15.11 | 74 | 0.39 |  | 14.29 | 70 | 0.38 |
|  | ACC | 8.17 | 40 | 0.21 |  | 8.58 | 42 | 0.23 |
|  |  |  |  |  |  |  |  |  |
| Val | GTG | 2.86 | 14 | 0.10 |  | 2.45 | 12 | 0.09 |
|  | GTA | 12.25 | 60 | 0.43 |  | 11.23 | 55 | 0.42 |
|  | GTT | 10.82 | 53 | 0.38 |  | 10.21 | 50 | 0.38 |
|  | GTC | 2.65 | 13 | 0.09 |  | 2.86 | 14 | 0.11 |
|  |  |  |  |  |  |  |  |  |
| Trp | TGG | 5.10 | 25 | 0.25 |  | 7.56 | 37 | 0.36 |
|  | TGA | 15.72 | 77 | 0.75 |  | 13.48 | 66 | 0.64 |
|  |  |  |  |  |  |  |  |  |
| Tyr | TAT | 41.45 | 203 | 0.81 |  | 43.29 | 212 | 0.73 |
|  | TAC | 10.00 | 49 | 0.19 |  | 16.13 | 79 | 0.27 |
|  |  |  |  |  |  |  |  |  |
| End | TAG | 9.60 | 47 | 0.13 |  | 8.17 | 40 | 0.12 |
|  | TAA | 64.11 | 314 | 0.87 |  | 59.22 | 290 | 0.88 |
